# Acute-phase reactant protein profiles: an aid to monitoring large bowel cancer by CEA and serum enzymes.

**DOI:** 10.1038/bjc.1977.24

**Published:** 1977-02

**Authors:** A. M. Ward, E. H. Cooper, R. Turner, J. A. Anderson, A. M. Neville

## Abstract

The profiles of 4 acute-phase reactant proteins (APRPs) (haptoglobin (HPT), alpha1 antitrypsin (AAT), alpha1 acid glycoprotein (AGP) and prealbumin (PALB)) have been studied during the evolution of bowel cancer. Serial measurements of these APRPs can add to the information obtained from measurements of the level of CEA and hepatic enzymes during the monitoring of postoperative patients. There is considerable stability in the profile in a given individual in health, Rises of AAT and AGP are associated with metastases. High levels of HPT may suggest involvement of the bowel wall by recurrent cancer. PALB levels tend to reflect the nutritional status. A discriminant function based on the log CEA, AAT and AGP preoperative blood levels can considerably improve on the predictive value attained using CEA levels alone.


Br. J. Cancer (1977) 35, 170

### ACUTE-PHASE REACTANT PROTEIN PROFILES: AN AID TO MONITORING LARGE BOWEL CANCER BY CEA AND SERUM ENZYMES

A. MILFORD WARD,* E. H. COOPER,† R. TURNER,† J. A. ANDERSON‡ AND A. M. NEVILLE**

*From *The Protein Reference Unit, Hallamshire Hospital, Sheffield, †Department of Experimental Pathology and Cancer Research and ‡Department of Community Medicine, University of Leeds, and **Unit of Human Cancer Biology, The Ludwig Institute of Cancer Research, in conjunction with the Royal Marsden Hospital, Fulham Road, London SW3 6JB*

Received 9 August 1976 Accepted 11 October 1976

**Summary.—The profiles of 4 acute-phase reactant proteins (APRPs) (haptoglobin (HPT), $\alpha_1$ antitrypsin (AAT), $\alpha_1$ acid glycoprotein (AGP) and prealbumin (PALB)) have been studied during the evolution of bowel cancer. Serial measurements of these APRPs can add to the information obtained from measurements of the level of CEA and hepatic enzymes during the monitoring of postoperative patients. There is considerable stability in the profile in a given individual in health. Rises of AAT and AGP are associated with metastases. High levels of HPT may suggest involvement of the bowel wall by recurrent cancer. PALB levels tend to reflect the nutritional status. A discriminant function based on the log CEA, AAT and AGP preoperative blood levels can considerably improve on the predictive value attained using CEA levels alone.**

There is now a substantial body of evidence from Europe and North America that sequential measurement of the plasma carcinoembryonic antigen (CEA) concentrations can provide an earlier warning of the recurrence and metastasis of large bowel cancer (see Neville and Cooper, 1976 and Go, 1976 for reviews). However, this test alone has certain limitations, since CEA may not be elevated in primary tumours, especially Dukes A and B lesions, or when there is a minimal residual tumour load remaining after resection of the primary tumour. The probable site of the metastases is not indicated by the level of CEA, although this omission can be overcome, at least in part, by the synchronous measurement of serum enzymes that reflect disturbances of hepatic function (Cooper *et al.*, 1975; Munjal *et al.*, 1976; Schwartz, 1976) but these tests do not become positive until there is a considerable tumour load, as judged by inspection of the liver at laparotomy (Cooper *et al.*, 1976*a*). In a search for other biochemical tests that might be used to form an array for the monitoring of colorectal cancer, our attention has turned to the $\alpha$ globulins. In common with many forms of cancer, colorectal cancer often induces a disturbance of the $\alpha$ globulins, and an initial study of the behaviour of haptoglobin, a member of the $\alpha$ globulin group of plasma protein (Cooper *et al.*, 1976*b*), indicated that the change in the profile of several acute-phase reactant proteins (APRPs) warranted further study. In this paper we report changes in the profile of four APRPs, measured both in horizontal studies to relate the change of profile with evolution of the disease in individual patients, and in vertical studies to relate the general alterations encountered in various clinical stages to the natural history of the cancer and its metastases. This study does not attempt to mirror completely our total experience of CEA, which is now based on several thousand estimations in colorectal cancer. It has been designed, using selected test samples, to evaluate whether a profile of APRPs can provide information complimentary to CEA and hepatic function tests. We also describe how the application of a step-wise logistic discriminant analysis to the components of the array measured in pre-operative patients can provide a prognostic index that is more powerful than CEA alone.

### PATIENTS AND METHODS

*Patients*.—One hundred and two patients, aged 45–92 (mean 68) years, were studied. The 237 samples of blood tested included 70 taken preoperatively from patients at the time of their first presentation. The follow-up samples were selected from our serum bank, held at − 25°C, on the basis of the known pattern of evolution of the metastatic cancer, or evidence of potential cure as indicated by absence of evidence of recurrence and a normal CEA for 1–2 years after resection of the primary cancer. For convenience, the material has been stratified with respect to a number of clinical and laboratory-based criteria. Primary tumours were divided into: (1) those in which surgery was apparently successful (Dukes A, B and C) without any residual disease and patients have remained tumour free for at least one year; (2) apparently successful resections that subsequently developed evidence of metastases during a follow-up of 1–2 years; and (3) patients in whom the primary lesion was complicated by overt metastases which varied considerably in amount from minimal residual disease undetectable after the wound was closed to marked indicator lesions, especially in the liver.

The postoperative samples were grouped as: (a) tumour-free, in which there was no evidence of recurrence clinically and the CEA was $< 30$ ng/ml; (b) suspicious, in which the CEA was $> 30$ ng/ml but there was no evidence of tumour detected clinically; (c) pelvic and peritoneal metastases based on clinical findings; and (d) hepatic metastases that had either been observed at laparotomy or diagnosed subsequently as the result of a progressive elevation of CEA, associated with a rising $\gamma$ glutamyl transpeptidase and 5′ nucleotidase.

*Methods*.—The plasma CEA was measured using the assay described by Laurence *et al.* (1972), the upper limit of normal being taken as 15 ng/ml. A level of 30 ng/ml has been chosen as the decision point, since a rise above this value in a postoperative patient carries a probability of 0·95 that there is an underlying recurrent cancer. Gamma glutamyl transpeptidase (GGT) and 5′ nucleotidase (NTD) were measured as described previously (Cooper *et al.*, 1975).

Haptoglobin (HPT), $\alpha_1$-acid glycoprotein (AGP) and prealbumin (PALB) were measured by single radial immunodiffusion, using antisera and standard preparations obtained from Behringwerke AG, Marburg/Lahn, W. Germany. The HPT phenotypes were identified by electrophoresis on gradient pore polyacrylamide gels (Baxter and Rees, 1974). The correction factors applied were $\times$ 0·6 for Hp 1 : 1, $\times$ 1·3 for Hp 2 : 1 and $\times$1·5 for Hp 2 : 2. Antitrypsin (AAT) and ceruloplasmin (CPL) were measured by automated immunoprecipitation (Ritchie, 1973), antisera being obtained from Atlantic Antibodies Inc, Westbrook, Maine (AAT) and Behringwerke AG (CPL).

The normal range for these plasma proteins in our laboratories is HPT 0·5–2·5 g/l, AAT 1·8–3·0 g/l, AGP 0·6–1·0 g/l, PALB 0·2–0·4 g/l and CPL 0·1–0·3 g/l. These ranges are applicable to a healthy population over the age of 40 years, but do not necessarily apply directly to a population weighted in excess of 65 years, which would more closely approximate the cancer patients under study whose median age is 68 years.

*Statistical analysis*.—The grouped data are expressed as group means and the standard deviation (s.d.) and standard error (s.e.) of the mean. The discriminant function for the separation of patients who remained tumour-free for at least 1 year after surgery (Group I) from those who developed metastases after an apparently curative resection (Group 2) was derived by logistic discriminant analysis (Anderson, 1972).

A step-wise approach was taken to the establishment of the discriminant function. Thus the potential discriminators were considered one at a time, and the one ($\log_{10}$ CEA) which best separated the two groups was selected. Then the variable which best assisted $\log_{10}$ CEA to separate the groups was selected. This procedure continued, adding discriminant variables one at a time until the extra separation given by the next " best " discriminator was no longer statistically significant, as judged by an asymptotic log-likelihood ratio test. In the order selected by this procedure, the discriminant variables were $\log_{10}$ CEA, $\alpha_1$-antitrypsin and $\alpha_1$-acid glycoprotein, and the discriminant function was estimated to be:

$$I = 6{\cdot}2 - 2{\cdot}6x_1 - 1{\cdot}6x_2 + 1{\cdot}3x_3,$$

where $x_1 = \log_{10}$ CEA ng/ml, $x_2 =$ AAT g/l and $x_3 =$ AGP g/l as measured prior to surgery.

### RESULTS

### *Horizontal studies*

The behaviour of the profile of APRPs in colorectal cancer is best illustrated by considering their patterns during the follow-up after surgical treatment of the primary tumour. The following examples have been selected to indicate the chronological change in a battery of proteins which depicts graphically the events underlying the larger series that have been classified into a number of discrete events in the vertical studies.

Fig. 1 shows the evolution of the APRPs in a patient who developed hepatic metastases after an apparent disease-free interval. Six months prior to the start of monitoring, the patient had undergone a curative resection of a Dukes C1 tumour of the colon. During the course of the study the CEA rose from 26 to 125 ng/ml and the GGT was 60 iu/l at the last reading: subsequently the patient deteriorated rapidly, developing obvious clinical signs of hepatic metastases, and died 4 months after the last set of measurements. At the time of the last reading there had been no clinical evidence of metastases.

Fig. 2 shows the stability of the proteins after the 4th month following the resection of a Dukes B carcinoma of the colon in a woman aged 56 years. The first observation was made the day before surgery. At that time the CEA was 84 ng/ml and all the APRPs were raised. Subsequently the CEA has remained 10 ng/ml and the APRPs have been within normal limits.

Fig. 3 demonstrates the pattern of the APRP profile in a woman aged 72 years who had a Dukes B tumour of the recto-sigmoid excised in May 1973, and 9 months later was found to have a local recurrence that required excision. Subsequently she developed progressive spread of the tumour in the pelvis and peritoneal cavity, and died 7 months later. Plasma

FIG. 1.—Evolution of APRPs during the development of hepatic metastases. HPT, haptoglobin; AAT, α antitrypsin; AGP, acid glycoprotein.

FIG. 2.—Stability of the APRP profile in a patient who has remained free of recurrence. (For convenience the ceruloplasmin results (CPL) are × 10 to fit the scale.)

FIG. 3.—The disturbance of the APRP profile caused by recurrent tumour localized to the pelvis requiring a second resection. (CPL levels × 10.)

CEA gave a preoperative level of 26·8 ng/ml and 11 ng/ml immediately prior to excision of the recurrence, with a subsequent rise to 57 ng/ml 2 months prior to her death.

The APRPs show considerable variability in the immediate postoperative period and may take 1–3 months to reach a stable level, even in patients with potentially curable tumours whose postoperative course has been satisfactory. This is illustrated in Fig. 4. In our analyses we have excluded observations made in an immediate postoperative period. By contrast the postoperative fall in CEA is slightly faster, with stable values being reached 4–6 weeks after curative surgery.

*Vertical studies in postoperative patients*

The general evolution of the changes in the APRP, GGT and CEA levels are illustrated in Tables I and II. As indi-

FIG. 4.—Illustration of the effect of laparotomy and resection of the rectum. The initial values in this patient were all within normal limits.

cated in the methods section, the definition of a "normal" range is difficult—we consider that a good useful standard is best provided by the values found in postoperative patients in whom prolonged follow-up has proved that they are cured. This has been used to select the discriminant levels adopted in Table II. However, as emphasized above, it is the time course of the patterns that is usually more informative than the precise value. The tables are given as they illustrate the general applicability of the approach and condense the information into a convenient form. An analysis of the correlation matrix of these parameters shows that, although there are obvious linked trends, the correlation coefficients are not $> r = 0{\cdot}65$ in any pair combination, indicating that the system does not contain redundant information.

*Preoperative samples*

The distributions of the values of the components of the assay system are shown in Table III. Due to the marked skewness of the distribution of the CEA and GGT values in patients with metastatic cancer, the data have been transformed to their logarithms. The scatter can be appreciated from the increased s.d. of the GGT in the metastatic group compared to Groups 1 and 2. The mean of the CEA value rises progressively with the increase of the severity of the disease. The distribution of the CEA values in the three groups is shown in Fig. 5. It will be observed that the haptoglobin elevation

TABLE I.—*Postoperative Patients**

| | No. of pts. | No. of samples | HPT | AAT | AGP | PALB |
|---|---|---|---|---|---|---|
| Tumour-free ⩾ 2 years | 10 | 45 | 2·26† | 2·08 | 0·83 | 0·34 |
| | | | 1·08 | 0·52 | 0·25 | 0·12 |
| | | | 0·16 | 0·07 | 0·03 | 0·02 |
| Tumour-free transient | 11 | 18 | 2·87 | 2·35 | 0·99 | 0·32 |
| | | | 1·18 | 0·71 | 0·25 | 0·14 |
| | | | 0·28 | 0·16 | 0·06 | 0·03 |
| Suspicious (CEA > 30 ng/ml) | 6 | 12 | 3·89 | 2·92 | 1·37 | 0·25 |
| | | | 1·80 | 1·38 | 0·56 | 0·09 |
| | | | 0·32 | 0·38 | 0·12 | 0·02 |
| Hepatic metastases | 14 | 30 | 4·72 | 3·74 | 1·52 | 0·21 |
| | | | 1·80 | 1·43 | 0·74 | 0·12 |
| | | | 0·32 | 0·25 | 0·13 | 0·23 |
| Pelvic, peritoneal metastases and local recurrence | 22 | 54 | 3·38 | 2·57 | 1·13 | 0·23 |
| | | | 1·80 | 0·78 | 0·55 | 0·09 |
| | | | 0·23 | 0·09 | 0·08 | 0·01 |

* All values ⩾ 3 months after surgery.

† Mean, s.d., s.e. (g/l).

HPT, haptoglobin; AAT, $\alpha_1$ antitrypsin; AGP, $\alpha_1$ acid glycoprotein; PALB, prealbumin.

TABLE II.—*Percentage of Postoperative Samples Suggesting an Abnormality*

| | No. of samples | GGT† > 30 IU | CEA > 30 ng | HPT > 2·5 | HPT > 3·5 | AAT > 3·0 | AGP > 1·0 | PALB < 0·20 |
|---|---|---|---|---|---|---|---|---|
| *Tumour-free ⩾ 2 years | 45 | 11 | 0 | 29 | 22 | 5 | 12 | 15 |
| Tumour-free transient or suspicious | 30 | 40 | 40 | 57 | 47 | 33 | 57 | 30 |
| Pelvic, peritoneal and local recurrence | 53 | 13 | 26 | 75 | 40 | 25 | 48 | 42 |
| Hepatic metastases (early, GGT < 100 i.u./l) | 20 | — | 89 | 100 | 95 | 75 | 57 | 25 |
| late, GGT > 100 i.u./l) | 10 | — | 100 | 100 | 80 | 60 | 80 | 90 |

* 60% of these samples came from patients who subsequently developed metastatic cancer. The second and third sub-sets in Table I have been combined to simulate a population presenting at a follow-up clinic.
† GGT, γ glutamyl transpeptidase.

TABLE III.—*Preoperative Values of the Test Battery*

| | log GGT* | log CEA* | HPT | AAT | AGP | PALB |
|---|---|---|---|---|---|---|
| No recurrence | 1·3881 | 1·2543 | 3·89† | 2·65 | 1·12 | 0·18 |
| 1 year | 0·2817 | 0·3178 | 1·60 | 0·84 | 0·48 | 0·06 |
| $n = 37$ | 0·0468 | 0·0528 | 0·26 | 0·14 | 0·08 | 0·01 |
| Recurrence | 1·3391 | 1·7018 | 3·93 | 3·30 | 0·96 | 0·17 |
| 2 years | 0·2409 | 0·4950 | 1·40 | 0·85 | 0·36 | 0·06 |
| $n = 11$ | 0·0738 | 0·1529 | 0·44 | 0·26 | 0·11 | 0·02 |
| Metastases at | 1·5600 | 2·0859 | 4·33 | 3·87 | 1·46 | 0·17 |
| operation | 0·5323 | 1·013 | 1·69 | 1·03 | 0·89 | 0·09 |
| $n = 22$ | 0·1073 | 0·21260 | 0·35 | 0·22 | 0·19 | 0·02 |

* The antilog of these values gives the geometric mean, not the arithmetic mean (see Fig. 5 for the distribution of the CEA values).
† Mean, s.d., s.e. (g/l).

can be as marked in patients with a tumour with a good prognosis as in those in whom there was advanced metastatic cancer complicating the primary tumour. In a

FIG. 5.—Distribution of the preoperative $\log_{10}$ CEA values in the three groups of patients.

similar fashion, a low prealbumin was a common finding in all groups of primary cancers. The low values may reflect a poor nutritional status, which can complicate either a primary lesion or its metastases. The preoperative values of AAT and AGP were highest when the primary tumour was complicated by metastases: the pattern was similar to that seen when metastases developed following resection of the primary tumour.

Fig. 6 shows the extent to which the logistic discriminant function can separate the patients in Groups 1 and 2, and Fig. 7 demonstrates the application of the same function to the CEA, AAT and AGP values in patients found to have metastases at operation. The predictive value of these tests is summarized in the following statement.

When a " curative " operation was performed, 18 patients had a negative index, 8/18 (44%) recurred within 1–2

FIG. 6.—Separation of Groups I and II using the logistic discriminant.

FIG. 7.—Application of the logistic discriminant function to the preoperative values of Group III patients years, whilst only 3/27 (11%) with a positive index recurred in the same period; there were a further 3 patients with an index of 0, all of whom have remained without recurrence.

Similarly, of Group III patients, who had metastatic cancer at operation, varying from advanced disease to minimal residual cancer, 19/22 (86%) had a negative index. The probability of curative surgery diminished as the negative value of index increased. An index of $\geqslant 0$ was associated with a curative operation in 27/33 instances (82%); 5/6 of the failures with a positive discriminant had indices of $< 0{\cdot}5$. The advantage of the discriminant over the CEA alone as a prognostic factor can be seen by comparing the distribution of CEA (Fig. 5) with the corresponding discriminant values (Figs. 6 and 7). The advantage of the system lies in helping to allocate the CEA up to 100 ng/ml to the correct group. The value of GGT did not aid the discriminant, as high values were always associated with a high CEA. However, a negative discriminant with a raised GGT often increases the probability of finding hepatic metastases at laparotomy.

### DISCUSSION

The levels of various APRPs are frequently abnormal in cancer (see Koj, 1974 and Bacchus, 1975 for review). However, their reaction is non-specific: disturbances of various individual APRPs can be caused by injury, acute and chronic infection and various inflammatory and degenerative diseases. In the past, many vertical studies of individual components of the spectrum of APRPs have been studied as potential cancer tests and discarded. Even with the present increased precision of measurement, the APRPs alone are not a test for the presence of cancer. In bowel cancer this is especially true, as primary cancers may produce alterations of the APRPs that are closely mirrored by the responses to inflammatory diseases of the large bowel (Marner, Friborg and Simonsen 1975) so that the APRP profile does not help in the differential diagnosis of bowel cancer in which the CEA is normal or slightly raised, for this combination can be found in any of the inflammatory states. Nevertheless, gastrointestinal symptoms with a disturbed APRP profile, with or without an elevated CEA, are a strong indication for investigation of the gastrointestinal tract, and a persistent abnormality of the protein profile might be taken as an indication of a need to re-examine the bowel, if no lesion can be detected on the first x-ray studies. About 7% of colon tumours may not be detectable at the time of the first radiological examination (Lauer, Carlson and Wollaeger, 1965).

The relative stability of the protein profile in a given individual, as long as his health remains in steady state, is an essential property that makes protein profiling useful in cancer monitoring. The present studies have confirmed this stability, which was suggested by previous studies of HPT (Cooper *et al.*, 1976*b*). Random measurements of protein profiles after bowel surgery are of little value, other than that they may confirm suspicion of metastases that have been suggested by an elevated CEA. The profiling system only becomes a valuable indicator of tumour recurrence when it is measured sequentially. A similar experience has been reported by others using single or several APRPs (Mueller, Handschumacher and Wade, 1971; Vickers, 1974). We have found that APRPs can add to the information provided by CEA and liver enzyme markers for monitoring the chemotherapy of colorectal cancer (Bullen *et al.*, 1976). However, the APRPs have the disadvantage of being sensitive to signals produced by wounded tissues, so that stability may not be reached for 3 months after major surgery such as abdominoperineal excision of the rectum. AAT and AGP are both increased in metastatic cancer and tend to be load-dependent. This effect is also found in other forms of adenocarcinoma such as breast, stomach and transitional-cell tumours of the bladder (Milford Ward *et al.*, unpublished data). However, about 14% of the Caucasian population in the United Kingdom will be of a non-MAAT phenotype, which results in the levels of AAT being lower than normal (Cook, 1974).

HPT is a particularly sensitive indicator. High levels may accompany uncomplicated primary bowel cancer, the levels tend to remain above the normal range in patients who have had a resection, and are not influenced by the presence or absence of a colostomy. We have the impression, based on 4 patients, that an unexpected and sustained rise of HPT after resection of a colorectal cancer may indicate local recurrence or metastases involving the bowel wall.

PALB was added to the array as it is a negative reactant, and in longitudinal studies its slow fall may be the first sign of deterioration. PALB tends to take several months to reach its highest level in patients treated successfully, and also may be restored to the normal range when patients respond favourably to chemotherapy (Bullen *et al.*, 1976).

The combination of CEA and non-specific tumour markers may help in the identification of high-risk patients. It appears that, using a discriminant function based on CEA and APRPs, patients with a negative discriminant index have a worse prognosis than those in which it is positive. This is mainly, but not wholly, due to the value of the CEA. The ability of the discriminant to separate the probable failures from the probable successes in patients where the CEA is $< 100$ ng/ml is the main advantage of the system. It is well established that CEA values $> 100$ ng/ml carry a poor prognosis.

Current experience of CEA tests, and their use in combination with non-specific indicators, suggests that 2–3-monthly blood tests are needed if the system is going to be given a real chance to provide early warning. Obviously, high risk patients should be given priority, especially if the clinician intends to use the information as a possible indication for additional therapy, whilst the tumour load is low and the patient is in a good general state of health.

We wish to thank Mr J. Holmfield and Miss Sue Carter for their technical assistance and Miss J. Barlow for collating the clinical data. We are grateful for the cooperation of Professor J. C. Goligher, Mr R. Hall, Mr N. G. Graham, and Mr W. A. F. MacAdam for giving us the opportunity of investigating their patients.

Professor E. H. Cooper and Mr R. Turner are supported by the Yorkshire Cancer Research Campaign.

### REFERENCES

ANDERSON, J. A. (1972) Separate Sample Logistic Discrimination. *Biometrika*, **59**, 19.

BACCHUS, H. (1975) Serum Glycoproteins in Cancer. *Prog. clin. Path.*, **6**, 111.

BAXTER, S. J. & REES, B. (1974) Simultaneous Haptoglobin and Haemoglobin Typing of Blood and Blood Stains Using Gradient Polyacrylamide Gel Electrophoresis. *Med. Sc. Law*, **14**, 231.

BULLEN, B., COOPER, E. H., TURNER, R., NEVILLE, A. M., GILES, G. R. & HALL, R. (1977) Cancer Markers in Patients receiving Chemotherapy for Colo-rectal Cancer: A Preliminary Report. *Med. Ped. Oncol.* Submitted.

COOK, P. J. L. (1974) Genetic Aspects of the Pi System. *Postgrad. med. J.*, **50**, 362.

COOPER, E. H., TURNER, R., STEELE, L., NEVILLE, A. M. & MACKAY, A. M. (1975) The Contribution of Serum Enzymes and Carcinoembryonic Antigen to the Early Diagnosis of Metastatic Colo-rectal Cancer. *Br. J. Cancer*, **31**, 111.

COOPER, E. H., EAVES, G., TURNER, R., NEVILLE, A. M. & MILFORD-WARD, A. (1976*a*) Experience of Multiparametric Tests in the Monitoring of Large Bowel Cancer. *Bull. Cancer* (in press).

COOPER, E. H., TURNER, R., GEEKIE, A., NEVILLE, A. M., GOLIGHER, J. C., GRAHAM, N. G., GILES, G. R., HALL, R. & MACADAM, W. A. F. (1976*b*) Alpha Globulins in the Surveillance of Colo-rectal Cancer. *Biomedicine*, **24**, 171.

GO, V. L. W. (1976) Carcinoembryonic Antigen: Clinical Application. *Cancer, N.Y.*, **37**, 562.

KOJ, A. (1975) Acute phase reactants. In *Structure and Function of Plasma Proteins*, Vol. 1, A. C. Allison, Ed. London: Plenum Press, p. 73.

LAUER, J. D., CARLSON, H. C. & WOLLAEGER, E. E. (1965) Accuracy of Roentgenologic Examination in Detecting Carcinoma of the Colon. *Dis. Colon Rectum*, **8**, 190.

LAURENCE, D. R. J., STEVENS, U., BETTELHEIM, R. DARCY, D., LEESE, C., TUBERVILLE, C., ALEXANDER, P., JONES, E. W. & NEVILLE, A. M. (1972) Role of Carcinoembryonic Antigen. *Br. med. J.*, iii, 605.

MARNER, I. L., FRIBORG, S. & SIMONSEN, E. (1975) Disease Activity and Serum Proteins in Ulcerative Colitis. Immunochemical Quantitation. *Scand. J. Gastroenterol.*, **10**, 537.

MUELLER, W. K., HANDSCHUMACHER, R. & WADE, M. E. (1971) Serum Haptoglobin in Patients with Ovarian Malignancies. *Obstet. Gynaec.*, **38**, 427.

MUNJAL, D., CHAWLA, P. L., LOKICH, J. J. & ZAMCHECK, N. (1976) Carcinoembryonic Antigen and Phosphohexose Isomerase Glutamyl Transpeptidase and Lactic Dehydrogenase Levels in Patients with and without Liver Metastases. *Cancer, N.Y.*, **37**, 1800.

NEVILLE, A. M. & COOPER, E. H. (1976) Biochemical Monitoring of Cancer. *Ann. clin. Biochem.*, **13**, 283.

RITCHIE, R. F., ALPER, C. A., GRAVES, J., PEARSON, N. & LAWSON, C. (1973) Automated Quantitation of Proteins in Serum and Other Biological Fluids. *Am. J. clin. Path.*, **59**, 151.

SCHWARTZ, M. K. (1976) Laboratory Aids to Diagnosis—Enzymes. *Cancer*, **37**, 542.

VICKERS, M. (1974) Serum Haptoglobins: A Preoperative Detector of Metastatic Renal Carcinoma. *J. Urology*, **112**, 310.


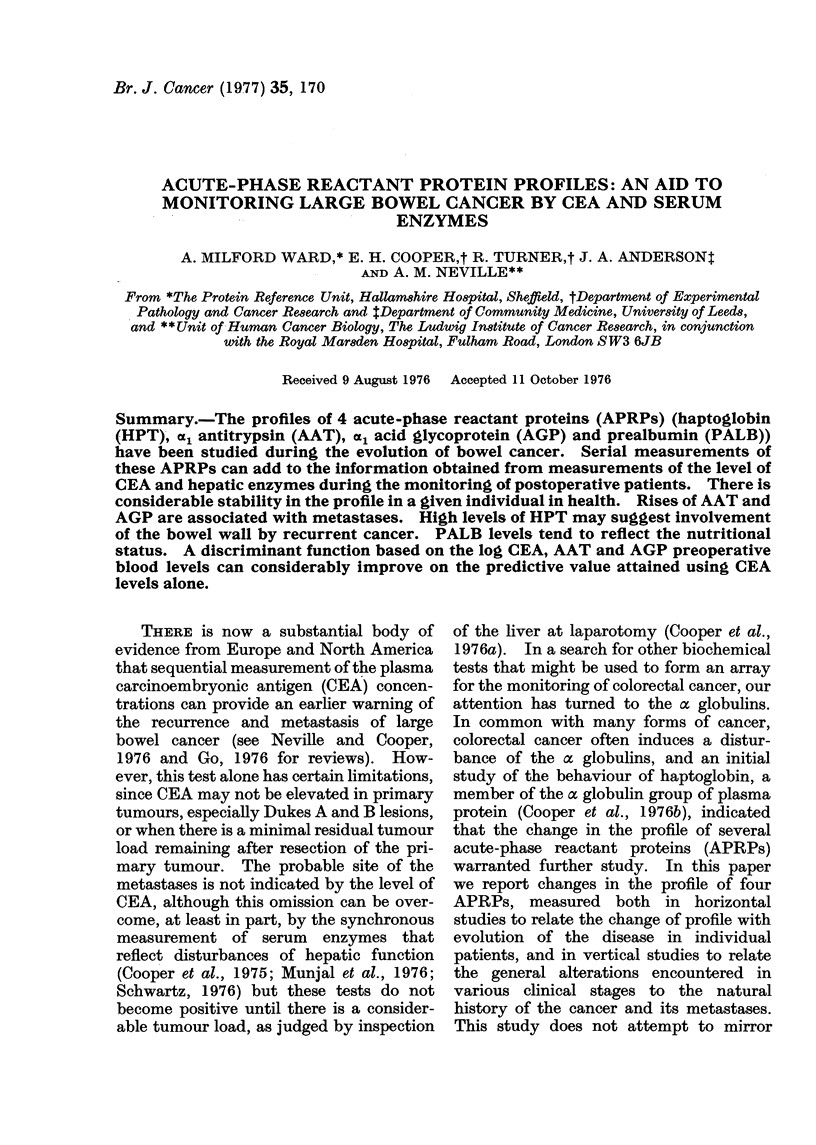

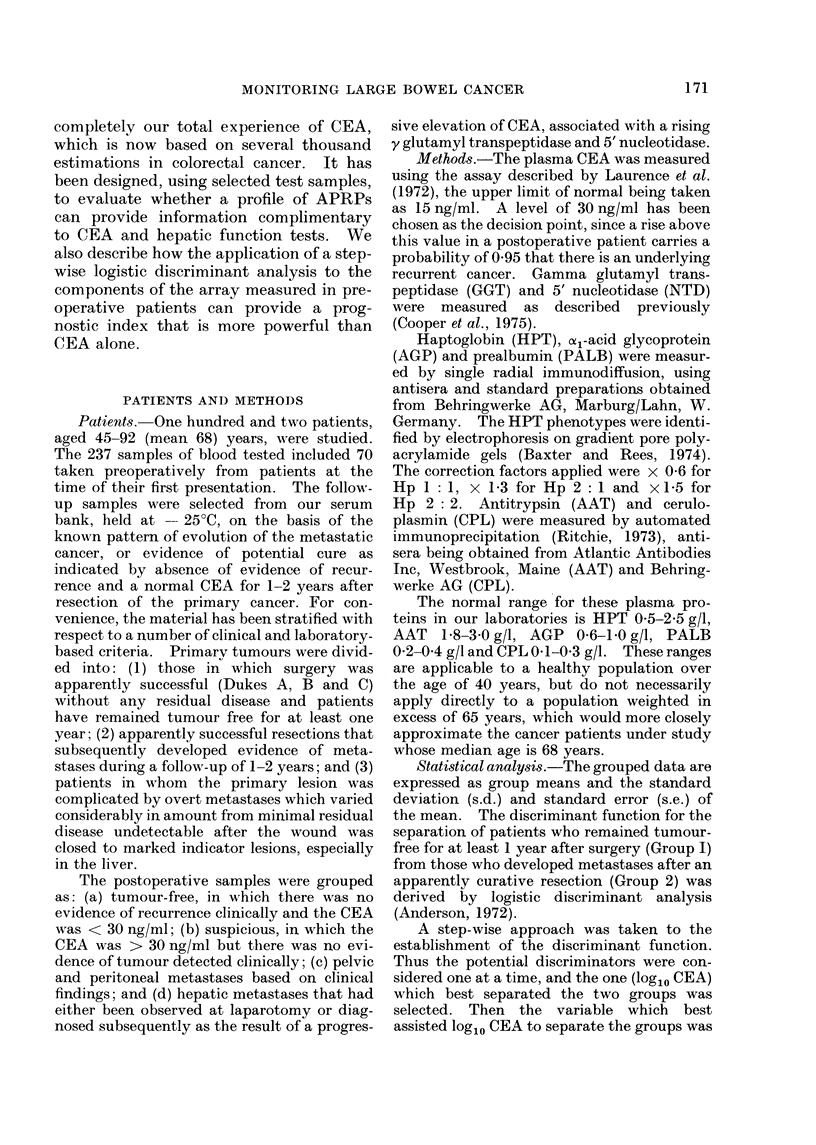

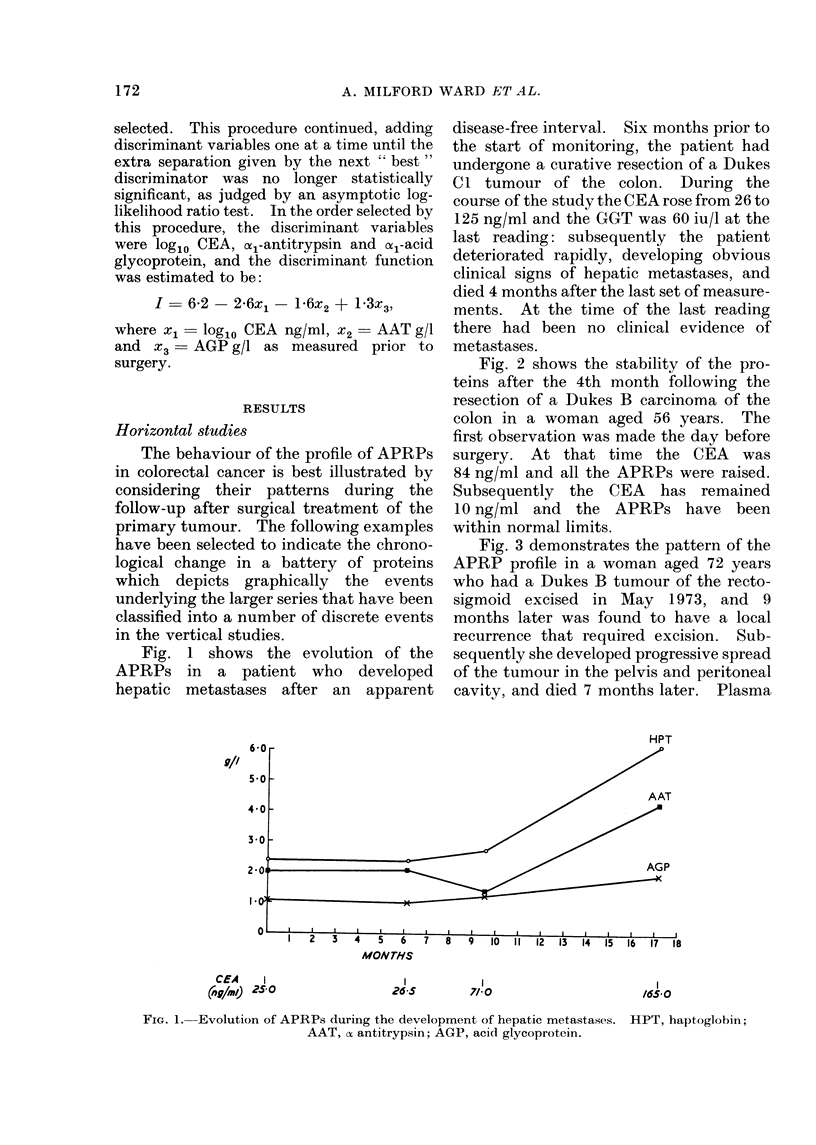

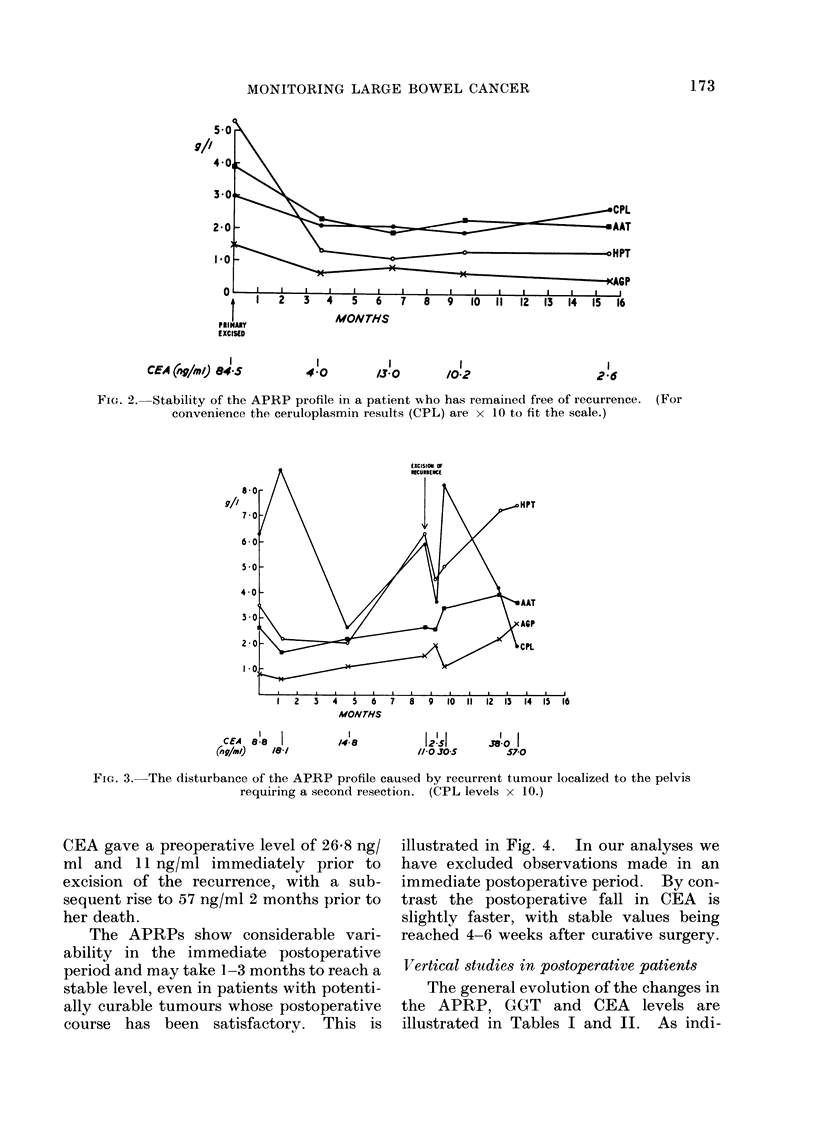

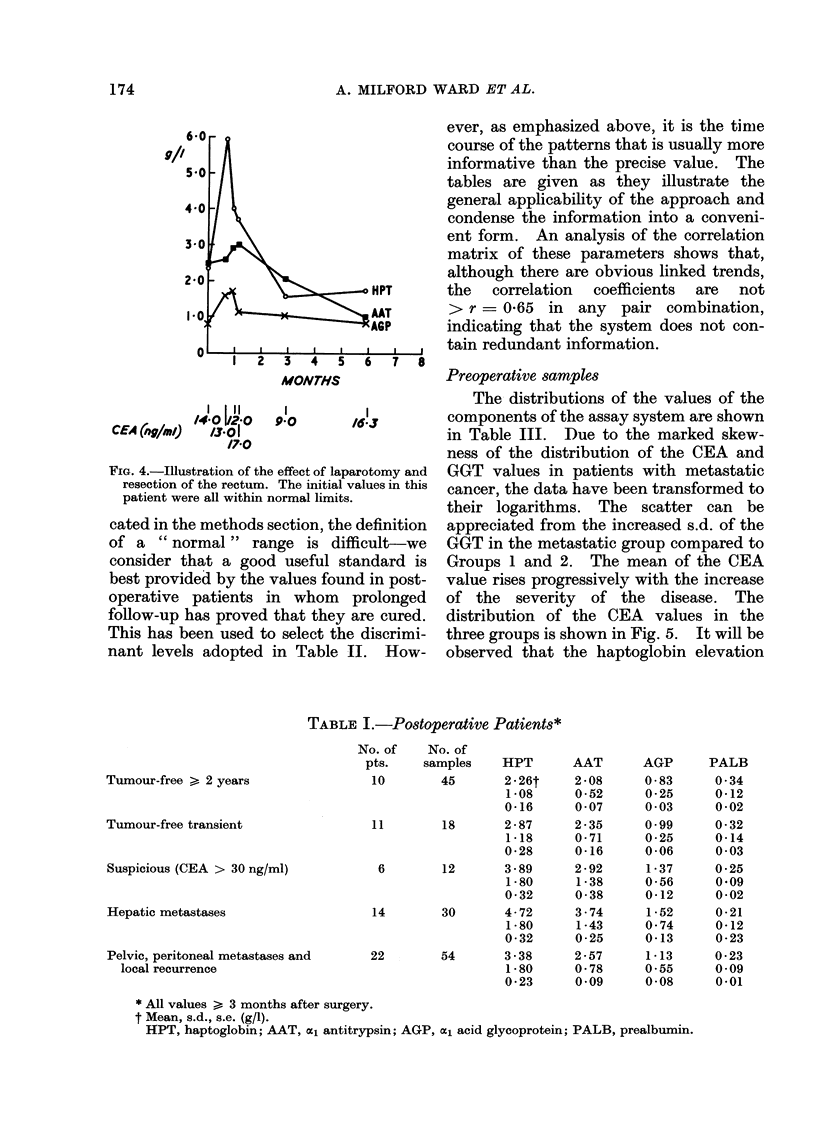

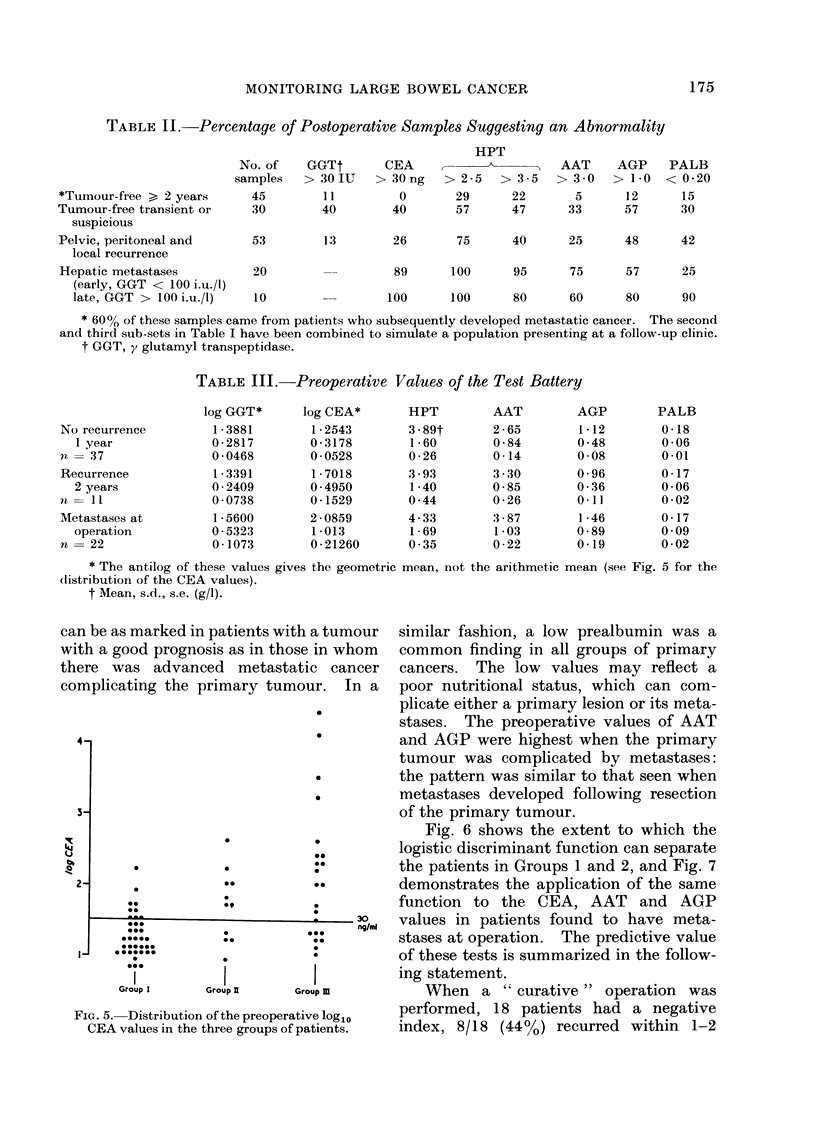

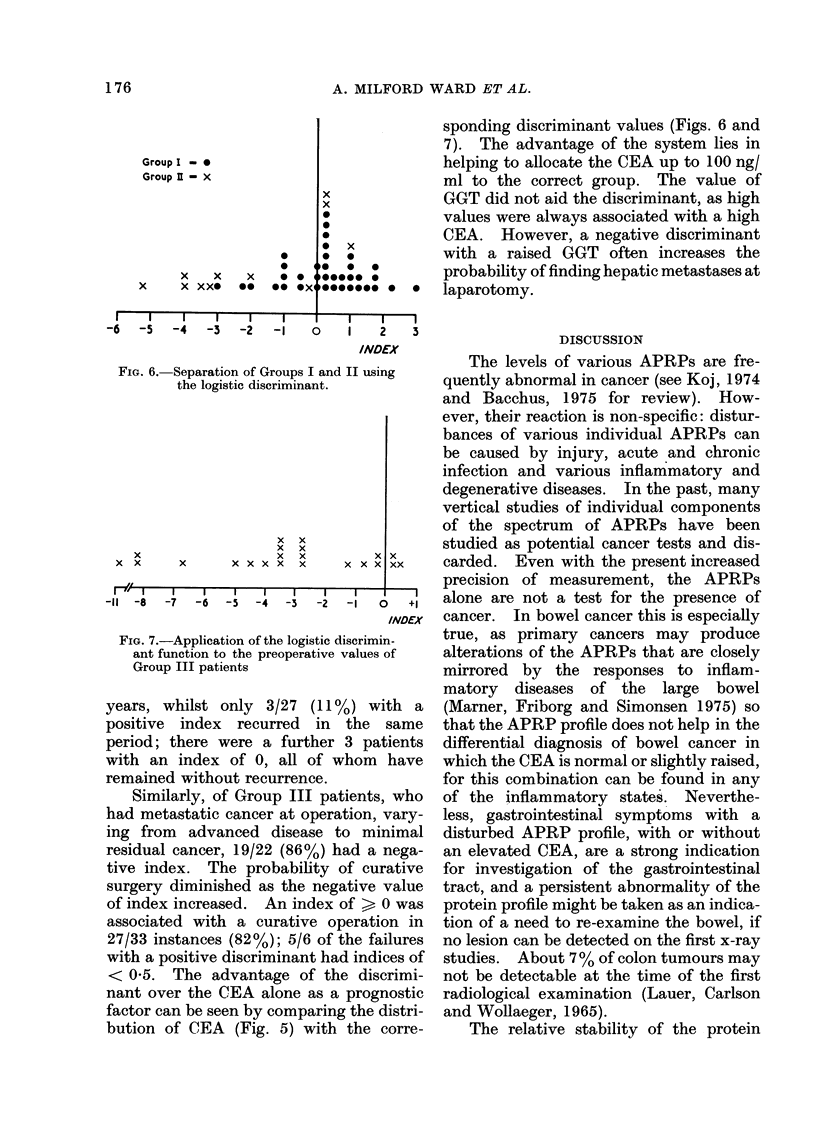

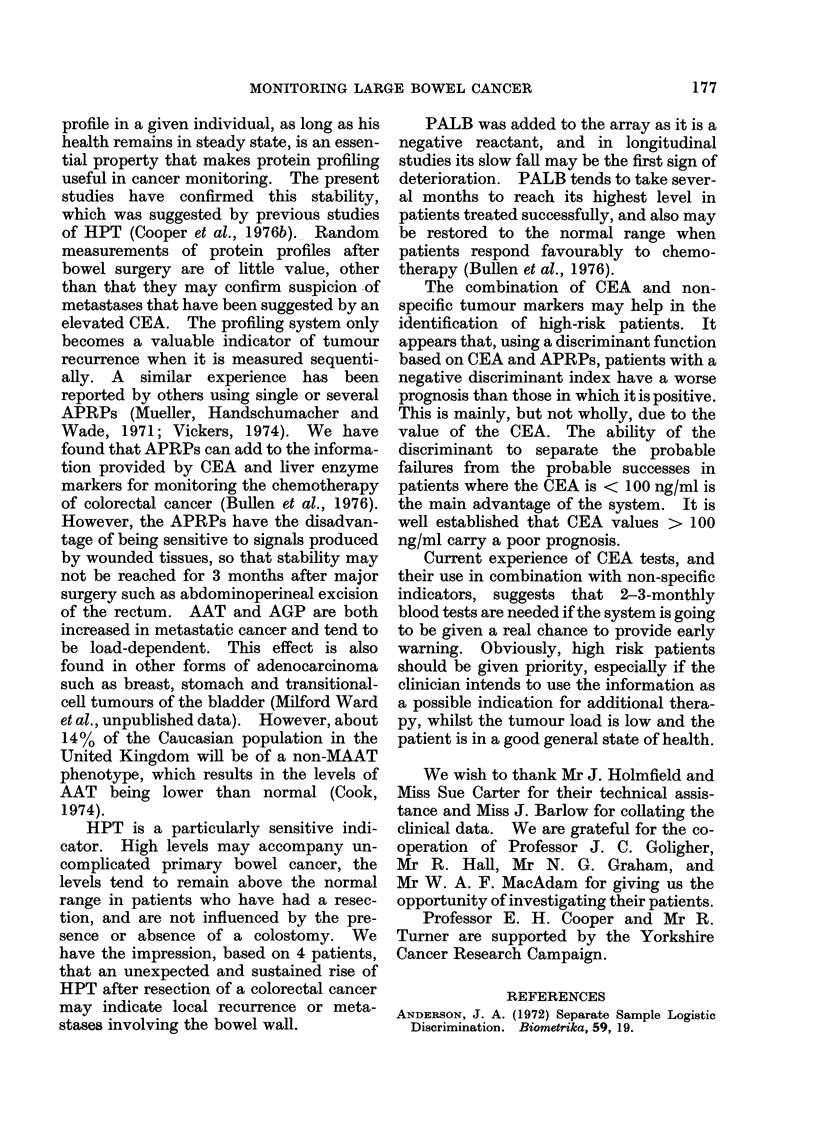

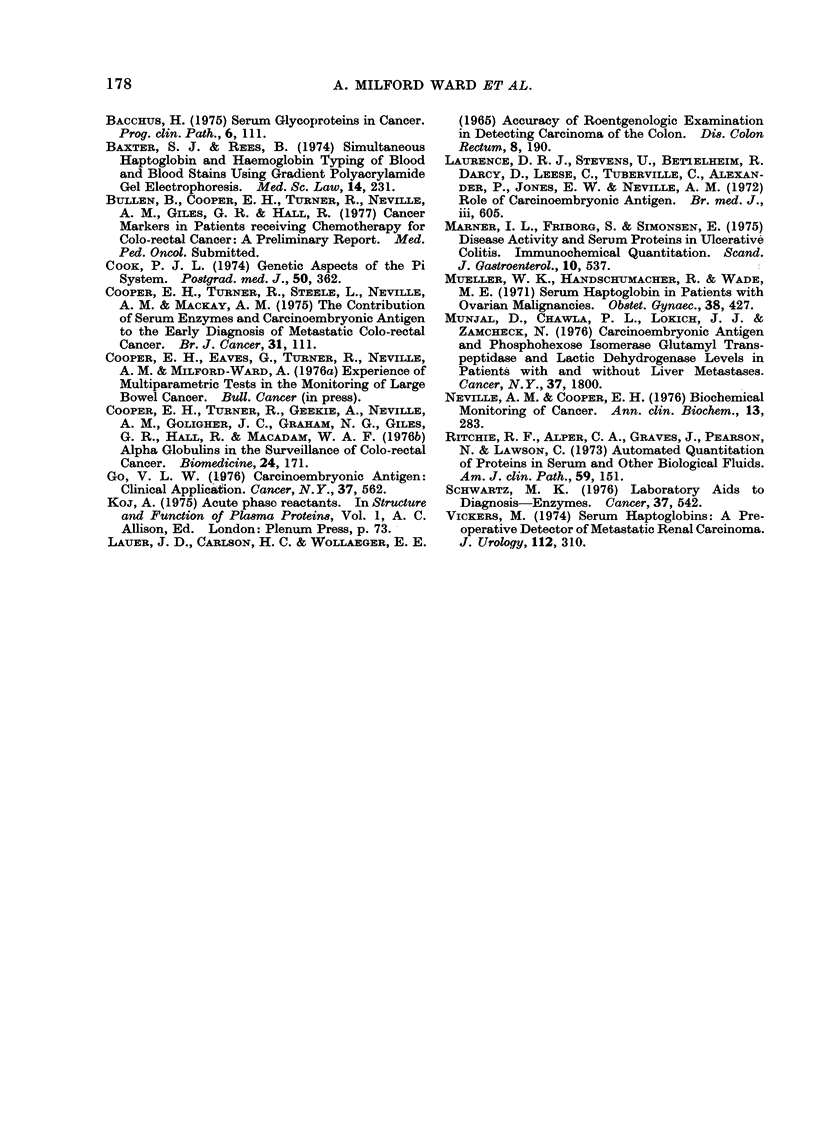


## References

[OCR_01034] Bacchus H. (1975). Serum glycoproteins in cancer.. Prog Clin Pathol.

[OCR_01038] Baxter S. J., Rees B. (1974). Simultaneous haptoglobin and haemoglobin typing of blood and bloodstains using gradient polyacrylamide gel electrophoresis.. Med Sci Law.

[OCR_01051] Cook P. J. (1974). Genetic aspects of the Pi system.. Postgrad Med J.

[OCR_01068] Cooper E. H., Turner R., Geekie A., Neville A. M., Goligher J. C., Graham N. G., Giles G. R., Hall R., Macadam W. A. (1976). Alpha-globulins in the surveillance of colorectal cancer.. Biomedicine.

[OCR_01055] Cooper E. H., Turner R., Steele L., Neville A. M., Mackay A. M. (1975). The contribution of serum enzymes and carcinoembryonic antigen to the early diagnosis of metastatic colorectal cancer.. Br J Cancer.

[OCR_01084] LAUER J. D., CARLSON H. C., WOLLAEGER E. E. (1965). ACCURACY OF ROENTGENOLOGIC EXAMINATION IN DETECTING CARCINOMA OF THE COLON.. Dis Colon Rectum.

[OCR_01093] Laurence D. J., Stevens U., Bettelheim R., Darcy D., Leese C., Turberville C., Alexander P., Johns E. W., Neville A. M. (1972). Role of plasma carcinoembryonic antigen in diagnosis of gastrointestinal, mammary, and bronchial carcinoma.. Br Med J.

[OCR_01097] Marner I. L., Friborg S., Simonsen E. (1975). Disease activity and serum proteins in ulcerative colitis. Immunochemical quantitation.. Scand J Gastroenterol.

[OCR_01103] Mueller W. K., Handschumacher R., Wade M. E. (1971). Serum haptoglobin in patients with ovarian malignancies.. Obstet Gynecol.

[OCR_01108] Munjal D., Chawla P. L., Lokich J. J., Zamcheck N. (1976). Carcinoembryonic antigen and phosphohexose isomerase, gammaglutamyl transpeptidase and lactate dehydorgenase levels in patients with and without liver metastases.. Cancer.

[OCR_01116] Neville A. M., Cooper E. H. (1976). Biochemical monitoring of cancer. A review.. Ann Clin Biochem.

[OCR_01121] Ritchie R. F., Alper C. A., Graves J., Pearson N., Larson C. (1973). Automated quantitation of proteins in serum and other biologic fluids.. Am J Clin Pathol.

[OCR_01127] Schwartz M. K. (1976). Laboratory aids to diagnosis--enzymes.. Cancer.

[OCR_01131] Vickers M. (1974). Serum haptoglobins: a preoperative detector of metastatic renal carcinoma.. J Urol.

